# Identification of an Oxygen Defect in Hexagonal Boron Nitride

**DOI:** 10.1021/acs.jpclett.2c02687

**Published:** 2022-10-06

**Authors:** Song Li, Adam Gali

**Affiliations:** † Wigner Research Centre for Physics, Post Office Box 49, H-1525Budapest, Hungary; ‡ Department of Atomic Physics, Institute of Physics, Budapest University of Technology and Economics, Műegyetem rakpart 3, H-1111Budapest, Hungary

## Abstract

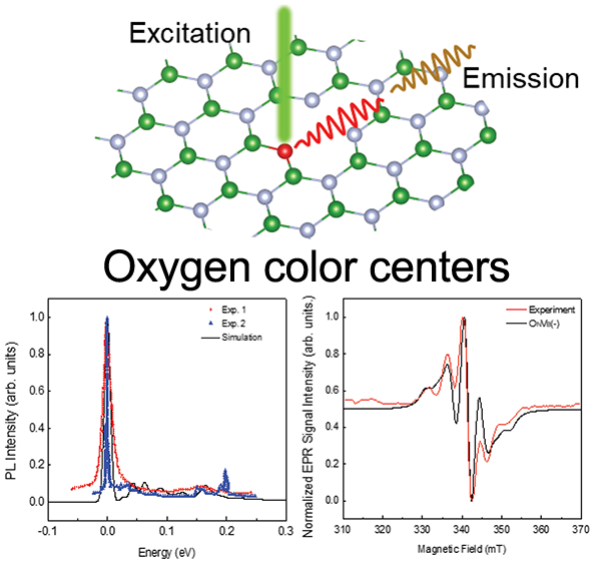

Paramagnetic fluorescent defects in two-dimensional hexagonal boron nitride (hBN) are promising building blocks for quantum information processing. Although numerous defect-related single-photon sources and a few quantum bits have been found, except for the boron vacancy, their identification is still elusive. Here, we demonstrate that the comparison of experimental and first-principles simulated electron paramagnetic resonance (EPR) spectra is a powerful tool for defect identification in hBN, and first-principles modeling is inevitable in this process as a result of the dense nuclear spin environment of hBN. In particular, a recently observed EPR center is associated with the negatively charged oxygen vacancy complex by means of the many-body perturbation theory method on top of hybrid density functional calculations. To our surprise, the negatively charged oxygen vacancy complex produces a coherent emission around 2 eV with a well-reproducing previously recorded photoluminescence spectrum of some quantum emitters, according to our calculations.

Optically active quantum defects at room temperature in two-dimensional (2D) hexagonal boron nitride (hBN) have emerged as an outstanding building block in quantum technologies covering nanoscale sensing, computing, and information processing.^1−8^ As a result of the large band gap of host hBN (about 6 eV), many point defects can induce multiple in-gap bound states with energy spacing in a wide spectral region from infrared to ultraviolet, without any interference from the ambient environment. The multiple in-gap bound states can be often optically active upon illumination. Spatially well-isolated fluorescent point defects, formed either spontaneously or by engineering, act as single-photon emitters with representing an ideal two-level system.^9,10^ In comparison to the three-dimensional (3D) host, e.g., diamond or silicon carbide, the low dimensionality of hBN does not introduce unwanted surface spin noise^11,12^ and enables high quantum efficiency (high light extraction ability). In addition, together with the diverse growth techniques, deterministic creation of defects has been achieved in atomic precision, which is an inevitable step toward solving the scalability issue required for quantum technologies.^13−15^ Recent experiments also realized the integration of defects with other quantum architectures.^13^ These advantages of 2D defects endow them with desirable key features, including high brightness, narrow photoluminescence (PL) line shape, emission tuned by strain,^5,16,17^ and electric fields.^18^

Up to now, strong emission signals have been recorded in the experiment from the ultraviolet (UV) region with zero-phonon-line (ZPL) energy at 4.1 eV^6,19−21^ and in the visible region at 1.6–2.2 eV.^1−4,8,22,23^ Various types of point defects have been proposed and assigned to single-photon emitters, including native defects, like boron vacancy (V_B_),^24−26^ nitrogen vacancy (V_N_),^27,28^ and Stone–Wales (SW) defects,^29,30^ external impurities, especially carbon defects,^3,4,27,31−34^ and more complex systems, such as donor–acceptor pairs with various impurities.^32,35^

Identification of these quantum emitters is extremely difficult. Although high-resolution transmission electron microscopy (HRTEM) images can provide information about the defect structures on the top layer of hBN,^5,36,37^ no one-to-one correspondence to the observed quantum emitters can be provided because the typical spatial resolution by optical means is about 100 nm and the quantum emitters may be found in deeper regions of multilayer hBN rather than the top layer. Optical signals alone provide very little direct information about the chemical nature of the defect, although the ZPL and the photoluminescence line shape, i.e., the phonon sideband (PSB), can be principally computed on the defect models by first-principles calculations (e.g., ref (38)). However, these computation methods have not yet achieved the chemical accuracy to identify the quantum emitters based on these properties alone. Furthermore, the absolute position of the ZPL may also shift with the environment in the experiments (e.g., strain); thus, it has become very challenging to identify the defects by only comparing the observed and computed PL spectra. We note that the isotope shift in the PL spectra may provide indirect information about the chemical nature of the defect;^34,39^ however, these types of investigations are still in infancy in hBN.

In this Letter, we propose another strategy to effectively identify defects in hBN. Paramagnetic defects can be observed by electron paramagnetic resonance (EPR) studies with producing a very characteristic spectrum as a result of the interaction between the electron spin and the neighbor nuclear spins, called the hyperfine interaction. The so-called Fermi contact term in the hyperfine interaction is associated with the overlap of the spin density with the nuclei, and its strength also depends upon the gyromagnetic constant of the given nucleus. As a consequence, the EPR spectrum is principally unique to the defect structure and provides direct information about the types of nuclei involved in the defect. It is a ground-state property of the defect that makes the modeling more simple than that for optical signals involving both the ground and excited states. Furthermore, the strength of the hyperfine interaction is generally not so sensitive to the environment as the optical signals. These considerations imply that spin-polarized density functional theory (DFT)-based methods can be very efficient to compute the ground-state spin density and the associated hyperfine tensors of defects in semiconductors.^40−42^ Unlike other hosts, such as diamond or silicon carbide, the 100% natural abundance of non-zero nuclear spin isotopes of hBN manifests a highly complicated hyperfine interaction, and its analysis is of vital importance in hBN.^43^ It is inevitable to compute the first-principles spin density distribution and derive the associated EPR spectrum for the interpretation of the experimental EPR data. This approach was already successfully applied for the negatively charged boron vacancy (V_B_) defect in hBN, which has a *S* = 1 ground state,^24^ and the electron spin resonance can be observed via optical means, i.e., optically detected magnetic resonance (ODMR) with coherent driving of ensembles,^2,25,44,45^ because the optical signal of V_B_ is intrinsically dim for single-spin observation.^25,26^ The hyperfine structure has been well-resolved in isotopically purified hBN samples and contributed to unambiguous identification.^46^ We note that understanding the hyperfine interaction is crucial in the decoherence processes^46^ and controlling^47^ the spin defects in hBN, to realize electron–nuclear entanglement acting as quantum memory and quantum register.^48−53^ Unfortunately, very few EPR spectra have been reported in hBN with often non-conclusive origin (see ref (3) and references therein). Very recently, a novel EPR center has been observed with a very characteristic hyperfine pattern,^54^ which provides a hope for unambiguous identification of a defect in hBN, as recently demonstrated for the V_B_ defect in hBN.

In this Letter, we employ plane-wave supercell DFT and many-body perturbation method calculations on the spin density distribution and hyperfine interactions of defects and identify the negatively charged oxygen vacancy defect as the origin of a novel EPR center in hBN. We further determine its PL spectrum and optical lifetime, and to our surprise, we find excellent agreement with the properties of some previously observed quantum emitters. We also evaluate the potential application of the defect as a qubit. Our results here not only provide valuable information for single-photon source identification in hBN but a proposal for quantum information processing in a 2D material.

Our spin-polarized DFT calculations are carried out using the Vienna *ab initio* simulation package (VASP) code.^55,56^ Projector augmented wave (PAW) formalism^57,58^ is used together with the plane wave basis set to describe the valence electrons and core nuclei. The monolayer hBN model is not sufficient to describe the interlayer screening effect between hBN sheets; therefore, to include the interlayer interaction, we use a bulk model with two hBN layers and periodic boundary conditions. The DFT-D3 method of Grimme for dispersion correction is used.^59^ An 8 × 8 supercell with 256 atoms is constructed to avoid the artificial defect–defect interaction. The structure is fully relaxed with the convergence force threshold at 0.01 eV/Å, and the plane wave cutoff is set to 450 eV. The screened hybrid density functional of Heyd, Scuseria, and Ernzerhof (HSE)^60^ is used to optimize the structure and calculate the electronic properties. In this approach, we could mix the nonlocal Hartree–Fock exchange to the generalized gradient approximation of Perdew, Burke, and Ernzerhof (PBE) with fraction α. α = 0.32 reproduces the experimental band gap at about 6 eV with the caveat that our method does not include the electron–phonon-related renormalization energy.^61^ The single Γ-point scheme is convergent for the sampling of the Brillouin zone. The excited states were calculated by the delta self-consistent field (ΔSCF) method.^62^ Charge correction is needed for the charged system in the supercell formalism, and it is computed by the SXDEFECTALIGN code.^63^ The EPR simulation is performed with EASYSPIN software at the X band region (9.45 GHz).^64^ The non-radiative recombination is computed with NONRAD.^65^ The self-consistent many-body perturbation theory based on the quasiparticle self-consistent *GW* (QPGW) method^66,67^ is applied to compute highly accurate quasiparticle wave functions and spin density distributions. Because of the high computational demands of the many-body perturbation theory method, a limited 5 × 5 monolayer model is used with more than 150 empty bands included. The energy cutoff is set to 250 eV. The wave functions and quasiparticle energies are updated until the convergence has been achieved. Typically, five iterations in the QPGW cycle are sufficient for this particular defect on top of initial HSE DFT calculations.

Our focus is on the hyperfine interaction, which is expressed as *H* = ∑_*i*,*j*_*Ŝ*_*i*_*A*_*ij*_^*N*^*Î*_*j*_, where *A*_*ij*_ is the hyperfine tensor and *Ŝ*_*i*_ and *Î*_*j*_ are the electron spin and nuclear spin, respectively. The tensor can be separated by two parts, the isotopic Fermi contact term and anisotropic dipolar term, as
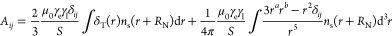
1where *n*_s_(*r* + *R*_N_) is the electron spin density, *r* is the vector between the electron spin and nuclear *R*_N_, γ_e_ and γ_I_ are electron and nuclear gyromagnetic ratios, respectively, and δ_T_(*r*) is a smearing function, which is discussed in detail in ref (68). We note that the hyperfine interaction requires an accurate calculation of the spin density at the place of nuclei. In principal, the all-electron PAW method enables that which modifies eq 1 within the PAW formalism.^40^ The effect of spin polarization of the core states in the Fermi contact term can be taken into account perturbatively, as implemented in VASP.^40^ The hyperfine tensor can be diagonalized, and the three principal values, i.e., hyperfine constants, are labeled as *A*_*xx*_, *A*_*yy*_, and *A*_*zz*_, where *A*_*zz*_ is chosen to be the largest absolute value that follows the convention in the EPR community.

Here, we discuss a recently discovered EPR center in hBN,^54,69^ which has been observed by absorbing the microwave photons in a X-band (∼9.45 GHz) cavity. The electron spin resonance signal of the spin triplet V_B_^–^ was observed by the ODMR technique,^2,44,69^ and we simulated the associated EPR signal in the X band, as shown in Figure 1. Recently, three spin doublet EPR centers generated by neutron irradiation of hBN have been reported. However, only one of them, labeled as D3, exhibits a characteristic hyperfine splitting pattern;^54^ thus, we focus our study on the D3 EPR center only. The splitting pattern demonstrates five peaks with intensities of 1:2:3:2:1. This indicates that the electron spin (*S* = ^1^/_2_) interacts with two equivalent nitrogen ^14^N nuclear spins (*I* = 1). The strength of the hyperfine interaction (∼100 MHz) implies that two nitrogen dangling bonds are involved in the defect. A natural model could be envisioned by starting with the V_B_ defect with three nitrogen dangling bonds and then either passivating the third dangling bond by hydrogen or replacing one nitrogen atom with an impurity atom “X”, i.e., V_B_ + X_N_ defect, which removes the nitrogen dangling bond. The hydrogen passivation model is in conflict with the observed EPR data because hydrogen is of almost 100% natural abundance and no hydrogen-related hyperfine splitting was detected. In the V_B_ + X_N_ model, the impurity atom should have very low natural abundance of nuclear spin isotopes, for example, carbon (^13^C = 1.1%), oxygen (^17^O = 0.037%), silicon (^29^Si = 4.7%), or sulfur (^33^S = 0.67%). One carbon-related defect, known as C_N_V_B_^–^, has been tentatively assigned to the D3 EPR center based on a common assumption that carbon is a frequent impurity in hBN. However, our theoretical study indicates that C_N_V_B_^–^ does not retain the *C*_2*v*_ structure with two nitrogen dangling bonds but rather quickly relaxes to a Stone–Wales-like configuration without any energy barrier.^70^ The carbon atom forms a bond to one of the three nitrogen atoms, which reconfigures to a pentagon ring that leaves only one nitrogen dangling bond behind. This configuration together with the spin density distribution leads to a relatively weak hyperfine interaction, and the calculated hyperfine constants are not consistent with the observed hyperfine constants (see Table 1). Hence, the carbon impurity is unlikely to be associated with the experimental EPR signal. Silicon and other heavy elements are too large and would form a bond with the other two nitrogen atoms, as shown in Figure S1 of the Supporting Information. Sulfur is not a common impurity in hBN and can be disregarded. Overall, there is compelling evidence that oxygen is involved in the D3 EPR center, and we use it as a working model in the context.

**Figure 1:**
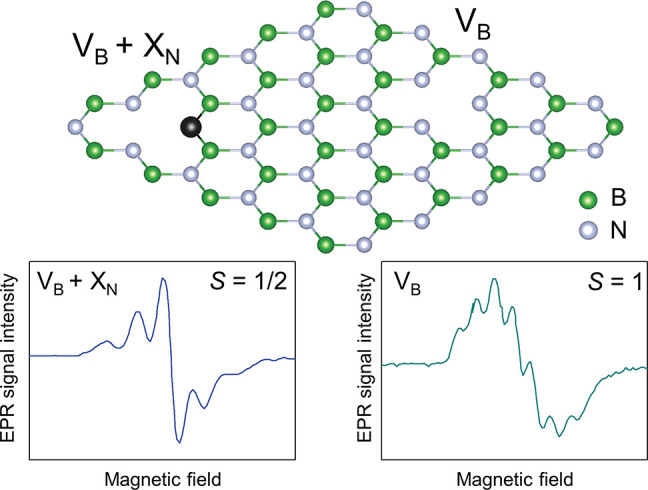
Schematic view of the V_B_ + X_N_ defect that is responsible for the observed EPR signal with a five-line splitting pattern^54^ and V_B_ defect with a seven-line splitting pattern.^2^ X indicates an external impurity atom.

**Table 1:** Hyperfine Constants of the Nearest Nitrogen Nuclear Spins in the D3 EPR Center and Two Defects

	configuration	*A*_*xx*_ (MHz)	*A*_*yy*_ (MHz)	*A*_*zz*_ (MHz)
^14^N	experiment^54^	90–98	90–98	145–148
^14^N	V_B_O_N_^–^	82.78	86.22	151.73
^14^N	sw-C_N_V_B_^–^^70^	6.75	6.53	13.25

We first briefly discuss the stability of the V_B_ + O_N_ defect. A pure single oxygen substitution defect like on the boron site (O_B_) and nitrogen site (O_N_) has been studied before.^31,71,72^ O_B_ has overly high formation energy compared to O_N_; consequently, the latter one could dominate with a large concentration.^31^ O_N_ is a doublet (*S* = ^1^/_2_) in the neutral charge state, and the occupied defect state is at 0.3 eV below the conduction band minimum (CBM); therefore, it is a hyper-deep donor. This donor could form a complex with the native acceptor boron vacancy (V_B_), i.e., the oxygen vacancy defect V_B_O_N_ shown in Figure 2a. The defect formation energy *E*_f_ is calculated to determine the charge stability as follows:
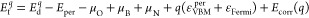
2where *E*_d_^*q*^ is the total energy of the hBN model with a defect at the *q* charge state, *E*_per_ is the total energy of the hBN layer without a defect, and μ_O_ is the chemical potential of oxygen and can be derived from a highly stable B_2_O_3_ crystal considering the environment of oxygen in hBN. Hence, μ_O_ is a function of μ_N_ as a good reference to future DFT calculations. The Fermi level ε_Fermi_ represents the chemical potential of the electron reservoir and is aligned to the valence band maximum (VBM) energy of perfect hBN, ε_VBM_^per^. *E*_corr_(*q*) is the correction term for the charged supercell as a result of the existence of electrostatic interactions with periodic conditions.

**Figure 2:**
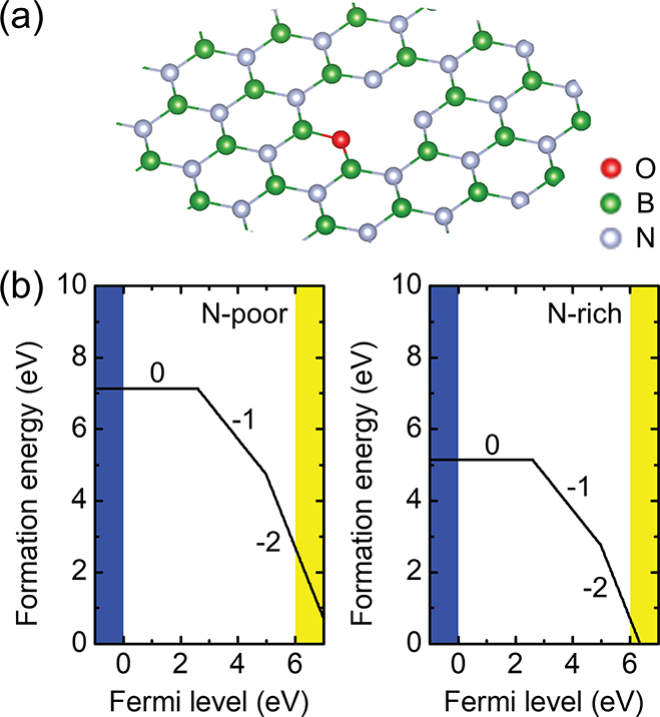
(a) Schematic view of the oxygen vacancy V_B_O_N_ defect in hBN. (b) Formation energy at N-rich and N-poor conditions and charge transition levels.

The calculated formation energy and charge transition level are plotted in Figure 2b. The positive charge state is not stable, and the ε(0/–1) level is at 2.61 eV. In the neutral charge state, the ground state could converge to the triplet (*S* = 1) state, as reported previously.^31^ The −1 charge state is a doublet (*S* = ^1^/_2_). The ε(−1/–2) level is at 4.96 eV; therefore, the −2 charge state requires heavy *n*-type doping. We employed a simple but a bit crude model for the chemical potential of oxygen; still the calculated formation energy is relatively low for the negatively charged configurations, which implies that the formation of the V_B_ + O_N_ defect is favorable.

We first discuss the −1 charge state, V_B_O_N_^–^, because it is spin-active with *S* = ^1^/_2_ and might be related to the observed EPR center. The energy level diagram is shown in Figure 3a. With *C*_2*v*_ symmetry, we can identify the irreducible representation of the in-gap defect states. Figure 3b plots the Kohn–Sham wave functions around the core of the defects. Low-energy *a*_2_ and *b*_2_ occupied levels reside just above the top of the valence band, which have similar Kohn–Sham energies. The highest occupied state in spin minority is *a*_1_, and its occupied counterpart falls in the valence band. Only one unoccupied state *b*_1_ appears in the spin-minority channel. The in-plane distribution of the *b*_1_ wave function (*p* orbitals) and, thus, the spin density is strongly localized on the two nitrogen atoms near the vacancy with producing large hyperfine constants, as listed in Table 1.

**Figure 3:**
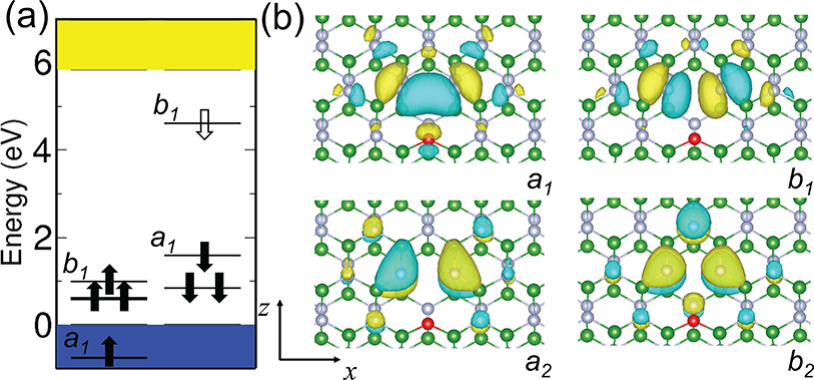
(a) Energy level diagram of V_B_O_N_^–^ in the ground state. The filled and empty arrows indicate the occupied and unoccupied defect states with spin-up and spin-down channels. The occupied *a*_1_ state falls into the valence band in the spin majority channel. The lowest optical excitation can be described by promoting an electron from the *a*_1_ state to the *b*_2_ state. (b) Wave function distribution of defect levels in the spin-down channel. The energy difference between *a*_2_ and *b*_2_ is negligible.

We note that we used the HSE results from the 8 × 8 bulk hBN supercell in Table 2. Although the HSE DFT method is considered to be accurate for the ground-state spin density of the defects, we go beyond DFT calculations and self-consistently updated the Kohn–Sham wave functions within QPGW many-body perturbation method calculations. This method is computationally very demanding; therefore, we compared the HSE and QPGW results in the 5 × 5 monolayer model. We find that the largest deviation for the critical hyperfine constants of nitrogen nuclear spins is less than 4 MHz, which is relatively tiny. We also found that the 8 × 8 supercell bulk hBN model is numerically well-converged; thus, our results are robust and provide direct evidence for the common assumption in defect modeling of hBN that the HSE DFT method works accurately for the ground-state properties, at least for the case of no highly correlated electronic structure, as provided by the V_B_O_N_^–^ defect.

**Table 2:** Convergence of the Calculated Hyperfine Constants of ^14^N Nuclear Spins with Various Sizes of Supercells^a^

size	*A*_*xx*_ (MHz)	*A*_*yy*_ (MHz)	*A*_*zz*_ (MHz)
5 × 5	79.65	83.02	148.73
5 × 5 SL	81.09	84.60	150.20
5 × 5 *GW* SL 150 eV	83.81	87.72	151.78
5 × 5 *GW* SL 250 eV	83.85	87.63	151.83
6 × 6	81.63	85.03	150.61
7 × 7	82.28	85.70	151.25
8 × 8	82.78	86.22	151.73
9 × 9	82.99	86.44	151.95

a We provide QPGW calculation in the 5 × 5 single-layer supercell labeled as 5 × 5 *GW* SL compared to the 5 × 5 HSE DFT single-layer data labeled as 5 × 5 SL. For the QPGW calculations we also provide the plane wave cutoff in eV unit used in the *GW* procedure. The other data are from HSE DFT calculations.

With the hyperfine tensor listed in Supplementary Note 1 of the Supporting Information, here, we simulate the ODMR in Figure 4b. A quintet splitting is clearly resolved as a result of the hyperfine coupling mainly from the two nitrogen atoms. We note that a limited amount of data is available to identify the subline width features. This requires low laser power and microwave power that minimize the broadening effect. To illustrate the challenge here, we quote a recent experiment that could identify double peaks in the ODMR spectrum of a single-spin system in hBN; however, they could not determine whether it is caused by a crystal-field effect in the high-field regime or hyperfine coupling.^73^ Several studies identified V_B_ with seven splitting peaks from the three identical ^14^N nuclear spins.^2,25,44,46^ A direct comparison shows that the ODMR spectrum of V_B_O_N_ is wider than that of V_B_ because the hyperfine constants of nitrogen nuclear spins in V_B_O_N_ are larger than those for V_B_ defect.^25^ The full width at half maximum (fwhm) of the central peak fitted through five Lorentzian functions is about 94 MHz for V_B_O_N_. We note that the estimated accuracy in our calculations by comparing the QPGW and DFT results (∼4 MHz) does not affect our conclusions. We argue that we identified the D3 EPR center as the negatively charged oxygen vacancy V_B_O_N_ defect.

**Figure 4:**
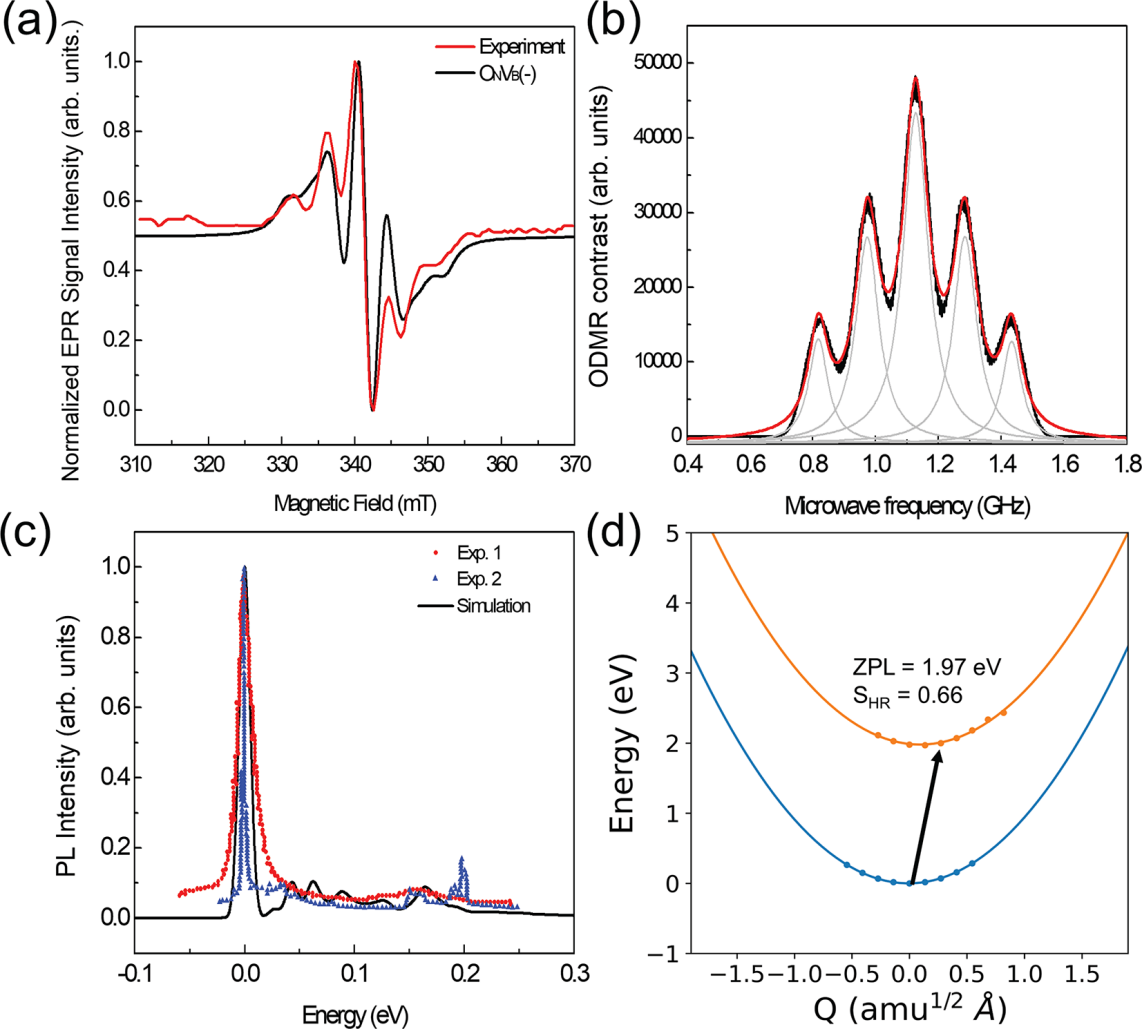
(a) EPR simulation with a X band at 9.45 GHz with the derivative absorption spectrum. As a result of the possibly tiny misalignment in the experiment, we slightly shift the simulation result by about 4 mT, which corresponds to about 9.5 GHz cavity frequency. The EPR spectra are recorded at room temperature in both the experiment and simulation. Both the experiment and simulation have Gaussian broadening of 0.8 mT and Lorentzian broadening of 3.3 mT. In simulation, the *g* factor of 2 is used. (b) Electron spin resonance absorption spectrum at a magnetic field of 40 mT, which may be observed by ODMR. The black line is the simulation result, and the gray lines are from Lorentzian fitting. A *g* factor of 2 is used, and the line width is set to 0.1 mT. (c) Simulated and experimental PL spectra. The ZPL energies are aligned to zero, to compare the PSB in the spectra. Experiment 1 was taken from ref (15) with ZPL at 2.16 eV, and experiment 2 is extracted from ref (29) with ZPL at 1.95 eV. (d) Configurational coordinate diagram of the ground and excited states for the non-radiative rate calculation.

The negatively charged V_B_O_N_ defect introduces both occupied and empty levels in the gap that is supposed to be optically active. Indeed, an electron may be promoted from the *a*_1_ state to the *b*_1_ state in the spin minority channel, which is an optically allowed transition. The calculated ZPL energy is at 1.97 eV, which is very close to that of the commonly observed quantum emitters at around 2 eV. The PSB is a key feature of the PL spectrum. Here, PSB simulation is based on Franck–Condon approximation, which can be described by the overlap between the phonon mode in ground and excited states.^38,62^ The calculated PSB well reproduces the observed feature at 165 meV, as shown in Figure 4c. The position of PSB corresponds to the native longitudinal optical phonon mode.^3^ The Huang–Rhys (HR) factor is 0.66, indicating a small electron–phonon coupling. Both *a*_1_ and *b*_1_ have an in-plane wave function distribution on the nearest neighbor two nitrogen atoms; therefore, the geometry of the ions has little changes going from the ground state to the excited state. We found two emitters with ZPL energies at 2.16 eV^15^ and 1.95 eV^29^ in the literature, which have very similar PL spectra. To our surprise, not only is the EPR center identified as V_B_O_N_^–^ but its optical signal was most likely observed as a bright single-photon emitter with strong coherent emission.

On the basis of the above result, the radiative lifetime can be evaluated as
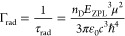
3where ε_0_ is the vacuum permittivity, ℏ is the reduced Planck constant, *c* is the speed of light, *n*_D_ = 2.5 is the refractive index of hBN at the ZPL energy *E*_ZPL_, and μ = 5.2 D is the optical transition dipole moment. The calculated radiative lifetime τ_rad_ is 11.8 ns. Besides the radiative recombination, non-radiative recombination may also occur. The non-radiative decay could couple with certain phonon modes and significantly alter the recombination mechanism and optical transition cycles (see a recent example in ref (74)). Here, we carefully consider the phonon-mediated non-radiative decay rate with Fermi’s golden rule. In Figure 4d, we map the adiabatic potential energy surfaces (APES) for the ground and excited states. The two states have very similar APES, and the effective vibriational frequencies *w*_i_ and *w*_f_ are about 88 meV. There is no crossing point between the two APES. In other words, the electron coupling is small (also reflected by the HR factor); therefore, the non-radiative decay is extremely slow, manifesting itself negligible, as shown in Table 3. Under this condition, the PL lifetime τ = τ_rad_ and we could achieve optimal quantum efficiency (near unity) if we exclude photoionization as an alternative non-radiative process. Defects with a relatively short PL lifetime have been observed in the region of 1–5 ns^1,8,16,75^ and also above 8 ns.^13,76^ We believe that V_B_O_N_ falls to the latter group. We note that the ODMR signal of half-integer single-spin systems has already been reported,^3,32,73^ which may also occur for the negatively charged V_B_O_N_ defect.

**Table 3:** Non-radiative Decay Parameters for V_B_O_N_^a^

*S*_HR_	Δ*Q* ( Å)	*W*_if_ (eV Å^–1^)	*w*_i,f_ (eV)
0.66	0.273	0.0037	0.088

a *S*_HR_ is the HR factor. Δ*Q* is the relative atomic displacement between excited and ground states. *W*_if_ is the electron–-phonon coupling matrix element. The calculation method is in Supplementary Note 2 of the Supporting Information.

Finally, it is worth noting the potential application of neutral V_B_O_N_ as a qubit with the triplet ground state. The zero field splitting is 2.36 GHz, which is smaller than that of V_B_.^25,44^ The energy level diagram is shown in Figure S3 of the Supporting Information. The optically allowed transition occurs between *b*_2_ and *a*_1_ defect states, and the corresponding radiative lifetime is 3.76 ms. The HR factor of this transition is 5.45. The reason for the relatively dim optical transition and large HR factor is that the *b*_2_ state is localized out of plane; therefore, it has a small overlap with the in-plane *a*_1_ wave function. As a consequence, the geometry relaxation is also huge between the two states. Unlike the negatively charged defect, the non-radiative decay cannot be ignored and our rough estimation yields a millisecond region, comparable to the radiative decay. Beside the radiative transition process and the above-mentioned non-radiative transition process, intersystem crossing (ISC) to a metastable singlet is another mechanism to decay from the excited state to the ground state. As a result of ISC, the fluorescence intensities are different between spin sublevels *m*_s_ = 0 and ±1. The initialization, manipulation, and readout are discussed in Supplementary Note 3 of the Supporting Information. These are the fundamental principles for the readout and initialization of the defect spin, which turns V_B_O_N_ to a qubit.

In conclusion, we demonstrated that the first-principles-simulated EPR spectrum can be well-employed to identify defects in a heavily dense nuclear spin environment when compared to the observed EPR spectrum. In particular, we performed spin-polarized DFT and many-body perturbation theory calculations on the V_B_O_N_ defect in hBN and found that the negatively charged defect is the so-called D3 EPR center. We showed that the defect was most likely observed as a quantum emitter with bright coherent emission at around 2.0 eV. Finally, we proposed a quantum protocol for neutral V_B_O_N_ to harness it as a qubit. Our present study provides an exciting result toward identification and manipulation of single defect spins^3,43,73^ in hBN.

## Supporting Information Available

Hyperfine tensor analysis (Supplementary Note 1), non-radiative decay calculation (Supplementary Note 2), and neutral V_B_O_N_ acting as a qubit (Supplementary Note 3) (PDF)
