# Supplementary material for: Identification of an Oxygen Defect in Hexagonal Boron Nitride
Source: J Phys Chem Lett. 2022 Oct 6;13(41):9544–51. doi: 10.1021/acs.jpclett.2c02687 (PMC9589898; doi:10.1021/acs.jpclett.2c02687)
Supplement: Supplementary file 1 — jz2c02687_si_001.pdf [file jz2c02687_si_001.pdf]

Supporting Information for Identification of an Oxygen Defect in Hexagonal Boron Nitride

Song Li† and Adam Gali*,†,‡

†*Wigner Research Centre for Physics, P.O. Box 49, H-1525 Budapest, Hungary*

‡*Department of Atomic Physics, Institute of Physics, Budapest University of Technology and Economics, Műegyetem rakpart 3., H-1111 Budapest, Hungary*

E-mail: gali.adam@wigner.hu

Table 1: Hyperfine constants of $V_BO_N(-)$. Here we consider the nuclear spin active isotopes (^{11}B, ^{14}N and ^{17}O). The unit is in MHz. The calculation is based on HSE with core polarization included. The polar (Θ) and azimuth (Φ) angle describing the eigenvector to A_{zz} are also given. $\Theta = 0$ means the eigenvector lies in hBN plane.

atom	A_{xx}	A_{yy}	A_{zz}	Θ	Φ
N113,121	82.79	86.23	151.75	90,90	145,85
N67,112	7.29	7.39	11.10	90,90	72,48
N105,122	6.32	6.45	9.84	90,90	163,103
N114	-1.44	-3.69	-4.08	0	-
B66,120	2.14	4.36	19.02	90,90	164,104
B113,121	-3.30	-11.45	-14.84	0,0	-,-
O	8.14	8.17	8.77	0	-

Supplementary Note 1: Hyperfine tensor

According to our previous study, the in-plane distribution wavefunction always yields large hyperfine coupling strength as shown in Tab. 1 that we use for ODMR simulation.

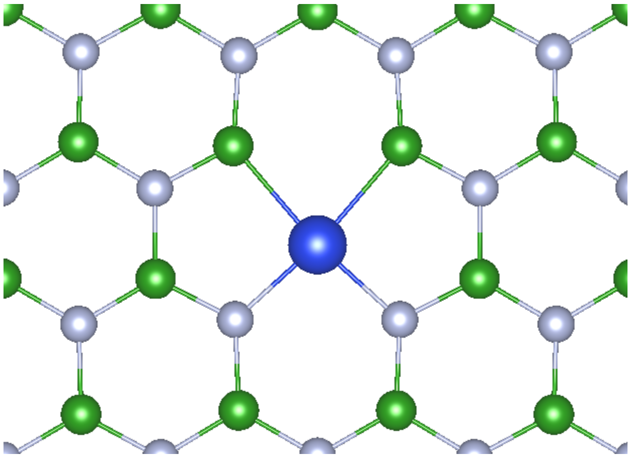

Figure 1: The optimized structure of silicon defect. The silicon occupies double vacancies, as Si_{VV}, and there is no dangling bonds from nitrogen atoms.

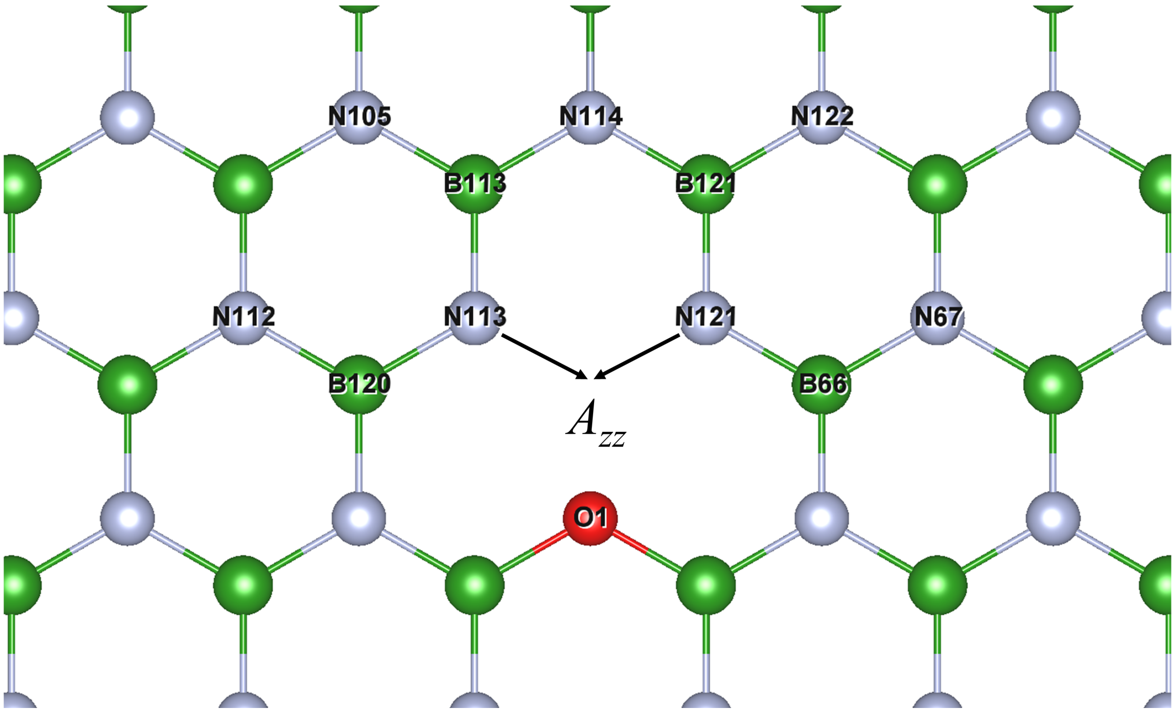

Figure 2: The atom label for hyperfine tensor in Table 1. The solid lines indicate the direction of hyperfine A_{zz} component.

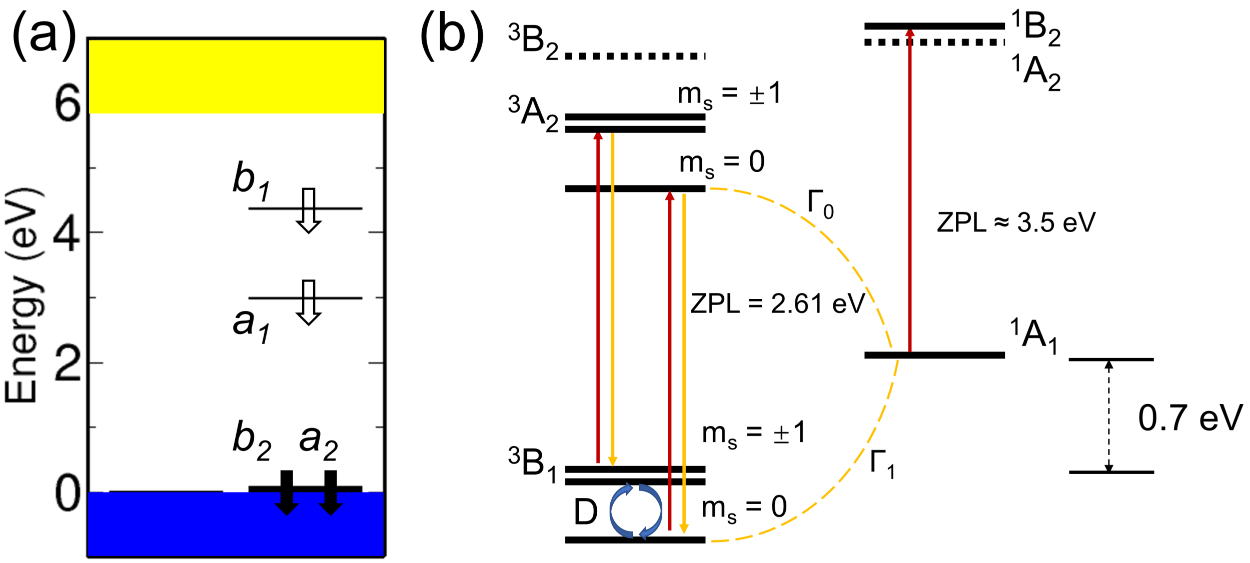

Figure 3: (a) The defect levels in gap of neutral V_BO_N triplet ground state. b_2 is lower than a_2 with energy difference about 0.04 eV. (b) The energy diagram of V_BO_N. Radiative and non-radiative transitions between ground state $^3B_1$ and excited state $^3A_2$ are depicted with red and yellow. The dashed lines indicate dark states with optical transition forbidden. There is ISC transition ($\Gamma_{0,1}$) between spin sublevel $m_s = 0$ in triplet and singlet states. Microwave pulse can be used to manipulate the qubit states at ground state.

Supplementary Note 2: Non-radiative decay calculation

The nonradiative transition rate can be calculated with Fermi's golden rule:

$$\Gamma_{\mathrm{nrad}} = \frac{2\pi}{\hbar} g W_{if}^2 X_{if}(T),$$

$$X_{if}(T) = \sum_{n,m} p_{in} \langle \chi_i | \hat{Q} - Q_0 | \chi_f \rangle^2 \times \delta(n\hbar\omega_i - m\hbar\omega_f + \Delta E_{if}),$$

$$W_{if} = \langle \psi_i | \partial_Q \hat{H} | \psi_f \rangle.$$

Here, W_{if} is the electronic term and $X_{if}(T)$ is temperature dependent phonon term. g is the equivalent energy-degenerate atomic configurations and p_{in} is the thermal population. i and f correspond the initial and final state. The phonon matrix $\langle \chi_i | \hat{Q} - Q_0 | \chi_f \rangle$ sums up the harmonic oscillator wavefunctions that enters the non-radiative recombination process. ΔE_{if} is energy difference between the two states and ψ is the single particle wavefunction from DFT.

Supplementary Note 3: Neutral $V_B O_N$ acting as qubit

The zero-field-splitting in the $S = 1$ ground state is 2.36 GHz which is smaller than that in V_B. The energy level diagram is shown in Fig. 3(a). There is no localized state in the gap in the spin majority channel and we find two occupied and unoccupied states in the spin minority channel. The a_2 to a_1 transition is forbidden so the allowed transition occurs between b_2 and a_1 defect levels and the corresponding radiative lifetime is 3.76 ms. Unlike for the negatively charged defect, the transition here is from out-of-plane orbital to in-plane orbital, and the HR factor of this transition is 5.45. The large HR factor indicates strong electron-phonon coupling, therefore the non-radiative decay cannot be ignored. We roughly estimate the non-radiative lifetime is also at millisecond region, comparable to the radiative process. Beside the radiative transition process and the above mentioned non-radiative one, intersystem crossing (ISC, Γ_0) to a metastable singlet is another mechanism for the decay of

the excited state back to ground state. However, accurate DFT calculation is problematic for open shell singlet calculation. More sophisticated methods beyond DFT are needed which is beyond our present scope. DFT predicts that the metastable singlet has an excitation energy at 3.57 eV which lies at higher energy than the optically allowed triplet $^3A_2$. Thus $^3A_2$ would directly relax to the singlet metastable state $^1A_1$. The transition rate highly depends on the spin-orbit interaction and the triplet-singlet energy gap. The electron then relax back to T_0 through Γ_1 and this is the optical initialization of qubit. The manipulation at T_0 can be achieved by resonance microwave between spin sublevels $m_s = 0$ and $m_s = \pm 1$. Due to the existence of ISC, the fluorescence intensities are different for the two spin sublevels, and this provide a way for readout the qubit states. However the calculated ZPL is at 2.61 eV which is close to the photoionization threshold energy. Further comprehensive study is needed to study the photostability of the neutral defect.
